# Steady and Dynamic Rheological Properties of Concentrated Chitosan Solutions at Different Concentrations and Temperatures

**DOI:** 10.3390/polym18151873

**Published:** 2026-07-30

**Authors:** Morteza Abazari, Soroush Yousefi, Soroush Barkhordari, Shahen Salih Mohammed, Mohammed Mahmood Ahmed, Safa Momeni Badeleh, Hossein Abdollahi

**Affiliations:** 1 Department of Pharmaceutical Nanotechnology, School of Pharmacy, Zanjan University of Medical Sciences, Zanjan 45139-56184, Iran; m.abazari119@gmail.com; 2 School of Pharmacy, Zanjan University of Medical Sciences, Zanjan 45139-56184, Iran; soroushyousefi834@gmail.com; 3 Department of Organic Chemistry, Faculty Chemistry, Alzahra University, Tehran 19938-93973, Iran; br_soroush@yahoo.com; 4 Department of Pharmaceutics, College of Pharmacy, University of Sulaimani, Sulaimani 46001, Iraq; shahen.ahmmed@univsul.edu.iq; 5 College of Medicine, University of Halabja, Halabja 46018, Iraq; muhamad.wallace@gmail.com; 6 Department of Food and Drug Control, School of Pharmacy, Zanjan University of Medical Sciences, Zanjan 45139-56184, Iran; safamo0568@gmail.com; 7 Department of Polymer Engineering, Faculty of Engineering, Urmia University, Urmia 57561-51818, Iran

**Keywords:** chitosan, rheology, viscosity, elastic, viscous, recoverability

## Abstract

The diverse properties and applications of chitosan materials require a broad spectrum of characterization techniques. In the meantime, due to chitosan’s high gel-forming capability, the rheological properties of chitosan solutions vary significantly depending on their state, i.e., solution or gel. To elucidate the solution and gel-based characteristics of chitosan, the present study was conducted to thoroughly investigate its rheological characteristics at different concentrations (1.25%, 2.5%, and 4% *w*/*v*), using various steady and dynamic rheological measurements and different mathematical models. The results showed that the concentration of chitosan solutions strongly affects their rheological properties in terms of shear stress, viscosity, and various dynamic rheological characteristics, through strain, angular frequency, and temperature sweep tests. In this regard, an elastic behavior was observed for low concentrations of chitosan solutions at lower shear strains, while the higher concentrations and higher shear strains exhibited predominantly viscous behavior. In frequency sweep measurements, the 1.25% and 2.5% *w*/*v* chitosan solutions showed viscous-like behavior and the 4% *w*/*v* solution exhibited solid-like behavior with almost constant storage and loss modulus profiles over the studied frequency range. According to temperature sweep measurements, viscous-like behavior was dominant at lower temperatures, which resulted in elastic gel structures at higher temperatures. Investigating the recoverability of prepared solutions by cyclic strain measurements revealed that the higher-concentration solutions of chitosan possess the desired capability to recover their structure. The results of this study provide detailed rheological insights into chitosan solutions suitable for designing and developing various chitosan-based products for different applications.

## 1. Introduction

Chitosan is a modified polysaccharide that consists of (1→4)-2-acetamido-2-deoxy-β-D-glucan (N-acetyl-D-glucosamine; NAG) and (1→4)-2-amino-2-deoxy-β-D-glucan (D-glucosamine) units linked by β (1→4) linkages and derived from carapaces of crustaceans and arachnids, such as shrimps, squids, and crabs. Chemically, chitosan is a partially crystalline and deacetylated biopolymer and contains two functional groups (hydroxyl and amine groups) in its polymeric backbone [1,2]. Owing to its diverse and valuable physicochemical and functional properties, such as biodegradability, biocompatibility, non-toxicity, and antibacterial activity, chitosan and chitosan-based materials and composites have found vast applications in various fields [3,4,5,6]. In this respect, chitosan-based materials and composites have been extensively studied and applied in various fields of science and technology, including medical, biomedical, pharmaceutical, packaging, textiles, and agricultural applications, among others [7,8,9]. In these areas, different aspects and characteristics of chitosan have been exploited to achieve the desired properties and satisfy the intended applications. Therefore, the wide-ranging physicochemical and functional properties of chitosan require a diverse spectrum of characterization techniques, and rheology measurements showcase one of the essential techniques with growing importance in this area [10,11].

Based on previous observations, the solubility of chitosan depends on both the degree of acetylation and molecular weight. Although low-molecular-weight chitosan and chitosan oligomers are soluble over a wide pH range from acidic to basic ones (i.e., physiological pH 7.4), chitosan samples with higher molecular weights are insoluble in organic and aqueous solvents with neutral and alkaline pHs and only can be dissolved in aqueous solutions of organic acids, such as formic acid, hydrochloric acid, and acetic acid at a pH below 6.2 due to protonation of the free amino groups present in its molecular structure [12,13,14,15,16]. Regarding this fact, the rheological and physicochemical properties of chitosan solutions depend on several parameters involved in its dissolution process. These parameters generally include the degree of deacetylation (DD), molecular weight, dispersity of macromolecules, concentration, type of acid, acid concentration, pH, ionic strength, counter ions, additives, temperature, and distribution of the acetyl groups along chitosan polymeric chains [17,18,19,20,21]. Moreover, like other natural polymers, chitosan has an amphiphilic nature originating from its hydrophilic amino groups, hydrophobic acetamido groups and the carbonaceous polymeric backbone of its molecular structure. As a result, the most physicochemical and rheological characteristics of chitosan materials in both solution and solid states depend on their ionic nature in the final product and the solution media. However, in contrast to most carbohydrate polymers, chitosan can also adopt strong positive charges due to the protonation of a great number of amino groups. This unique character of chitosan influences its behavior in solution states and endows this polymer with numerous practical and useful properties suitable for developing various single and composite products through blending with other polymers.

In addition to the above-mentioned functional and physicochemical properties, chitosan-based materials also showcase desirable processing characteristics, such as film-forming capability and form hydrogels when dissolved in an acidic environment. According to previous studies, increasing chitosan’s molecular weight, concentration, and DD, and the addition of physical and chemical crosslinking agents boost its gel-forming properties [22,23,24]. The rheological and physicochemical properties of chitosan in these formulations differ from those in their true solution states, and these formulations are generally considered a separate category in studying their various fundamental and practical characteristics.

Regarding the fabrication of chitosan products, wet techniques are usually employed, which involve preparing and processing chitosan solutions with varying viscosities, flowability, and characteristics. In order to prepare these solutions, the most common method is dispersing chitosan powder into acidic solutions and maintaining these solutions under continuous mixing for the required times to obtain homogenous solutions [25]. It is worth noting that these conditions do not constitute independent variables in the preparation of chitosan solutions, and it could be interesting to evaluate the impact of these conditions on the rheological properties of resultant solutions. Therefore, studying the solution-based characteristics and behavior of chitosan in both single and blended states is essential for designing and developing various products [25,26,27].

According to the generally accepted concept, rheology is the science of studying the deformation and flow of matter and describes the interrelation between force, deformation, and time [28]. In this sense, rheological studies provide valuable information about the flowability and viscoelastic characteristics, textural behavior of substances, macromolecule conformation, molecular relaxation phenomena, and structure–property relations between polymers and solvent systems [28,29]. Rheological measurements of polymeric solutions could be conducted through various steady-state or dynamic tests, and the different behaviors of solutions are usually described using various terminology, such as Newtonian and non-Newtonian (elastic or plastic) behavior, shear-thinning or shear-thickening character, thixotropic or rheopectic (time-dependent or time-independent) behavior, etc. The success of a wide range of commercial products and industrial processes depends on whether the specific rheological requirements of resultant polymeric solutions are met [28,29,30].

There are several works that have reported on the rheological properties of blended chitosan solutions and hydrogels with other natural and synthetic polymers [10,31,32,33], but few papers have been published on pure chitosan solutions. In one study by Kienzle-Sterzer et al., it was found that increasing the chitosan concentration increased the viscosity of resultant solutions, and at concentrations above 0.50 g/dL, shear-thinning behavior was observed. They also found that the zero-shear viscosity of chitosan solutions is independent of the ionic strength but dependent on the pH of the medium [34,35]. In another study, Wang and Xu found similar results for the shear stress and viscosity of chitosan solutions in relation to concentration and reported that higher DDs of chitosan result in non-Newtonian behavior [36]. In another survey, the effect of the temperature (20–50 °C) and chitosan concentration (2–10 g/L) on the viscosity of chitosan solutions was studied, and it was found that these solutions behaved like pseudoplastic fluids and that viscosity decreased as a function of the temperature and increased as a function of concentration [37]. The dynamic rheological characteristics of chitosan solutions with various concentrations (0.5, 1.0, 1.5, and 2.0%) at different temperatures (25, 30, 37, and 45 °C) and frequency (0.3–60 rad/s) were studied by Martínez-Ruvalcaba et al. The results showed that both the storage (G′) and loss (G″) moduli of solutions were elevated with increasing frequency. In all cases, G″ was higher than G’, but no crossover point was observed within the experimental range studied [38]. The effect of different organic and inorganic acids (lactic, acetic, formic, hydrochloric, lactobionic, mandelic acids, etc.) on the rheological properties of chitosan have been frequently studied in the previous literature, and the results showed that the different kinds of acids and their various concentrations (or different solution pH) affect the rheological behavior of chitosan in terms of viscosity, normal stress, G′, G″, etc. [14,39].

Despite many previous studies on the rheological properties of chitosan in both pure and blended states, as well as in various acidic media, a thorough rheological analysis of chitosan solutions has been scarcely investigated to date. Moreover, many of these studies have focused on the basic and viscosity-related measurements of chitosan-based hydrogels using low-molecular-weight chitosan materials, and the dynamic and advanced rheological studies of chitosan solutions have been barely evaluated in the previous literature. In this sense, the objectives of the present work were to thoroughly investigate the basic and dynamic rheological characteristics of chitosan solutions at different concentrations and temperatures, and to provide a detailed overview of the effective conditional and processing parameters that impact their rheological characteristics. Some mathematical models are also used in data analysis to correlate and describe the rheological data obtained for the studied solutions.

## 2. Experimental

### Materials and Reagents

Chitosan (medium molecular weight: 190–310 kDa, degree of deacetylation: 75–85%, Catalog number: 448877) and acetic acid (98%) were purchased from Merck (Darmstadt, Germany). All materials and reagents were used as received without further purification. Double-distilled water was used to prepare polymeric solutions for rheological studies.

## 3. Methods

### Preparation of Chitosan Solutions

Chitosan solutions with different concentrations (1.25%, 2.5%, and 4% *w*/*v*) were prepared by dissolving predetermined amounts of chitosan powder in 5% aqueous acetic acid at 85 °C under vigorous stirring for 12 h to obtain clear and homogenous solutions. The resultant solutions were subjected to rheological studies using different rheological experiments.

## 4. Rheological Studies

### 4.1. Steady Shear Measurements

A BROOKFIELD R/S-CPS+ RHEOMETER V. 9.00 (Brookfield Engineering Lab, Stoughton, MA, USA) with cone-and-plate geometry (CP75-1, diameter of 75 mm and a cone angle of 1°) was used to study the basic rheological properties and Flow measurements of the prepared solutions (such as shear viscosity and shear stress). Solutions were sheared continuously at a rate ranging from 0.01 to 300 s^−1^ at 25 °C with an equilibration time of 10 s at each shear rate to study the flow behavior and shear rate versus shear stress relationship. The shear stress vs. shear rate profiles of the solutions were analyzed by curve fitting procedures using the Ostwald–De Waele (1), Herschel–Bulkley (2), and Sisko models (3) using the following equations [40,41].

(1) $$\tau =K.{\dot{\gamma }}^{n-1}$$

(2) $$\tau =\tau_{0}+K.{\dot{\gamma }}^{n}$$

(3) $$\tau =K.{\dot{\gamma }}^{n}+\eta_{\infty }\dot{\gamma }$$

where τ represents the shear stress (Pa), $\dot{\gamma }$ is the shear rate (1/s), K is the consistency index (Pa.sn), n is the power law index, τ_0_ is the yield stress (Pa), and η_∞_ is the asymptotic value of the viscosity at very high or infinite shear rates (Pa.s). Similarly, the apparent viscosity profiles of solutions at different concentrations were further analyzed by fitting their corresponding experimental data to the following Power law (4), Cross (5), and Mizrahi–Berk (6) models [40,41,42].

(4) $$\eta_{a}=K.{\dot{\gamma }}^{-n}$$

(5) $$\eta_{a}=\eta_{\infty }+(\eta_{0}-\eta_{\infty })/1+(t\dot{\gamma }{)}^{n}$$

(6) $$\eta_{a}={(K}_{OM}+K_{M}{\dot{\gamma }}^{n}{)}^{2}/\dot{\gamma }$$

In these equations, η_a_ (Pa.s) is the apparent viscosity, $\dot{\gamma }$ (1/s) is the shear rate, n is the flow behavior index, η_∞_ (Pa.s) is the viscosity at infinite shear rate, η_0_ (Pa.s) is the zero-shear viscosity, t is the time constant (s^n^) and K_M_ and K_OM_ are consistency indices.

### 4.2. Dynamic Measurements of Viscoelastic Properties

For dynamic rheological studies and viscoelastic characterization of formulations, an advanced rheometer, Anton Paar, MCR300 (SN599139 model, Graz, Austria), equipped with a Peltier temperature-controlled system for fast and precise thermoregulation, was used. In this regard, the rheological characteristics of the formulations were assessed using amplitude sweep, frequency sweep, temperature sweep, and recovery tests. Amplitude strain experiments in the shear strain of 0.01–500% were conducted to obtain the linear viscoelastic (LVE) region and also determine the viscoelastic properties of solutions in terms of storage (G′) and loss modulus (G″), damping or loss factor (Tan δ), complex viscosity (η*), and shear stress profiles. The complex modulus (G*) of the solutions was calculated from their respective storage and loss modulus data sets using the following equation [43,44].

(7) $$G^{*}=\sqrt{G^{\prime 2}+{G\prime \prime }^{2}}$$

Frequency sweep measurements were performed from 0.01 to 100 rad/s at a constant shear strain of 10% and 24 °C to analyze the mechanical behavior of the formulations. To this end, various rheological characteristics of chitosan solutions, including dynamic moduli, damping factor, complex viscosity, and shear stress of the formulations, were investigated. The relaxation time (λ) under dynamic shear of solutions was calculated using the following equation [45,46,47,48].

(8) $$J^{\prime }=\frac{G^{\prime }}{([\eta^{*}]\varpi {)}^{2}}=\frac{\lambda }{\eta^{*}}$$

In this formula, J′ and ϖ are the complex compliance and angular frequency at each data point used during the rheological measurements, respectively. Temperature sweep experiments were conducted to study the gelation temperature of the solutions, ranging from 20 to 75 °C. Recoverability measurements were conducted using a cyclic strain test to assess the structural recovery of the formulations. The tests were carried out at oscillatory strain levels of 10% and 150%, with a constant angular frequency of 10 rad/s and a cycle duration of 10 s.

## 5. Result and Discussion

Due to the hygroscopic properties and high gelation ability of chitosan materials, preparing chitosan’s true solutions without any undissolved and agglomerated particles is challenging. The direct addition of chitosan powder to an aqueous acetic acid solution typically results in agglomerates, which will stay insolubilized, requiring a lengthy time of preparation and mixing process [49,50]. This may alter the concentration of the resultant solutions and produce chitosan solutions with different rheological and even physicochemical characteristics compared to the previously planned formulations. Moreover, as described in Section 1, the high gel-forming characteristics of chitosan-based materials lead to the formation of hydrogelic formulations that differ from their respective true solutions. On the other hand, preparing true and flowable chitosan solutions with the desired concentration and barrier effects is essential for developing specific end products and scaffolds, such as thin films, sheets, nanofibers, etc. [51,52,53].

**Figure 1:**
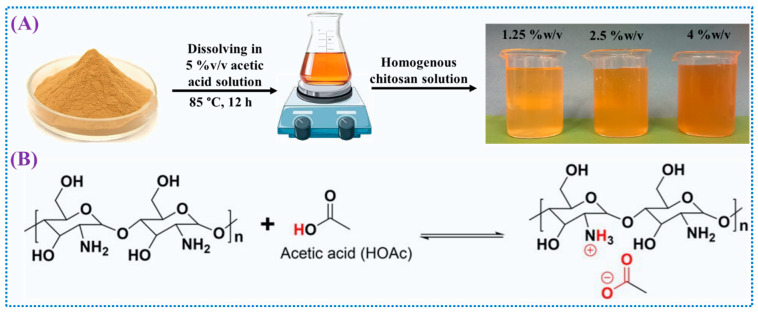
(**A**) Schematic representation of preparing chitosan solutions at different concentrations (1.25%, 2.5%, and 4% *w*/*v*). (**B**) Dissolution mechanism of chitosan in acetic acid solutions.

In this study, three concentrations of chitosan (1.25%, 2.5%, and 4% *w*/*v*) were prepared, ranging from dilute to concentrated, and their rheological characteristics were investigated. Due to its gelation at higher concentrations, chitosan solutions were prepared at concentrations up to 4% *w*/*v*. The three intended concentrations (1.25%, 2.5%, and 4% *w*/*v*) were selected to probe distinct regimes of chitosan solution behavior in accordance with classical polymer solution theory. At 1.25% *w*/*v*, the solution lies in the dilute regime, where individual chitosan chains are statistically isolated and chain–chain entanglement is minimal [54,55]. At 2.5% *w*/*v*, we target the semi-dilute entangled regime, where chain overlap, hydrogen bonding, and hydrophobic interactions can give rise to a transient network, reflected as nearly frequency-independent elastic behavior. Finally, 4% *w*/*v* represents a concentrated regime, in which overlap is extensive and larger clusters or microdomains may form, potentially giving rise to structural heterogeneity. Although these concentrations do not correspond to sharply defined boundaries (since the exact transitions depend on molecular weight, degree of deacetylation, and solution conditions), they were chosen to cover a scientifically and practically relevant window. From Figure 1, it is evident that all prepared solutions in this study were highly homogeneous and uniform, exhibiting a yellowish color and lacking any agglomerated or undissolved particles.

## 6. Steady Shear Measurements

**Figure 2:**
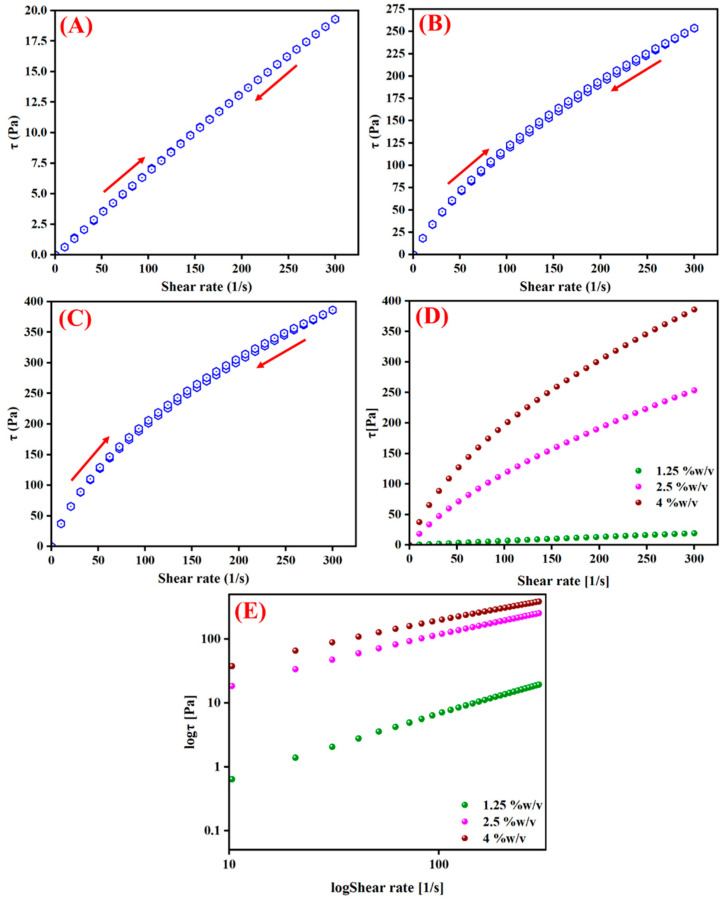
The forward and backward shear stress–shear rate profiles of chitosan solutions at (**A**) 1.25% *w*/*v*, (**B**) 2.5% *w*/*v*, and (**C**) 4% *w*/*v* concentrations. (**D**) Comparative forward shear stress–shear rate rheograms of chitosan solutions at different concentrations. (**E**) Double-logarithmic plot of forward shear stress–shear rate profiles of chitosan solutions at different concentrations.

To compare the basic rheological properties of chitosan solutions with different concentrations, the flow sweep measurements were conducted to obtain the two correlation curves [shear stress (σ) vs. shear rate ($\dot{\gamma }$) and viscosity (η) vs. shear rate ($\dot{\gamma }$)]. The forward and backward shear stress vs. shear rate profiles of solutions at 0.01–300 1/s shear rate range were shown in Figure 2A–C. It is evident that increasing the chitosan concentration significantly increases the maximum shear stress of solutions, from approximately 19.3 Pa for the 1.25% *w*/*v* solution to 253 and 386 Pa for 2.5% and 4% *w*/*v* solutions, respectively, at the maximum shear rate (300 1/s). The approximately linear relationship of shear stress–shear rate profile in 2.5% *w*/*v* solution (Newtonian or semi-Newtonian behavior) converted to a non-linear relationship as the total concentration of solutions increased (non-Newtonian behavior). This relationship between shear stress and shear rate is a characteristic behavior of polymeric solutions and represents a pseudoplastic or shear-thinning effect of the final solutions [37]. Moreover, it can be seen that as the concentration increases, the backward and forward rheograms of the solutions tend to separate from each other, especially at higher shear rates, forming a hysteresis loop. This hysteresis loop is another important characteristic of the non-Newtonian polymeric solutions. As indicated in Figure 2A–C, in all solutions, the backward rheograms are plotted under the forward rheograms, indicating a thixotropic behavior for prepared solutions [22].

**Figure 3:**
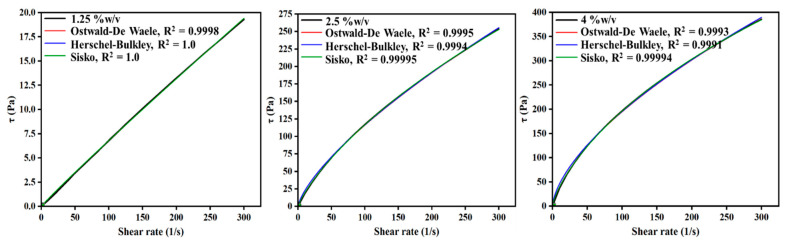
Curve fitting results of shear stress–shear rate forward rheograms of chitosan solutions at different concentrations (1.25%, 2.5%, and 4% *w*/*v*) using the Ostwald–De Waele, Herschel–Bulkley, and Sisko models.

The forward rheograms of all prepared solutions were extracted from their respective shear stress vs. shear rate profiles at different concentrations and the results in the form of normal and double logarithmic plots are depicted in Figure 2D,E. As mentioned above, the shear stress of the solutions increased in a concentration-dependent manner, with the highest shear stress recorded for solutions containing the highest amount of chitosan (4% *w*/*v*). A curve-fitting procedure using Ostwald–De Waele, Herschel–Bulkley, and Sisko models was applied to the data from shear stress–shear rate rheograms of each solution with different concentrations, and the results are depicted in Figure 3. From the linearities (R^2^) of each model in Figure 3, it is evident that all studied models successfully correlated with the obtained data for all studied solutions. However, the R^2^ values of the studied models slightly decreased with increasing the solution’s concentration. The numerical results of each model’s specific descriptors are summarized in Table 1. From this table, the consistency index obtained from different rheological models showed a concentration-dependent manner and significantly increased with increasing the solution’s concentration. The consistency index of polymeric solutions has been related to the intermolecular interactions and binding forces of their components, and higher K values indicate a stronger intermolecular interaction between polymeric molecules [56,57]. Therefore, the obtained results clearly imply higher interactions and binding forces for chitosan with higher concentrations. In contrast with the consistency index, the flow index “n” of solutions from all studied models consistently decreased with increasing the solution’s concentration. In this respect, both the Ostwald–De Waele and Herschel–Bulkley models exhibited identical values for all solutions, whereas the Sisko model produced higher values in contrast to the other two models. The flow or liquidity index values were correlated with the degree of pseudo-plasticity. In this context, “n < 1” refers to shear-thinning properties, while “n > 1” values indicate a shear-thickening character of solutions [58,59]. According to the results, the prepared solutions exhibited shear-thinning properties, which were significantly pronounced at elevated concentrations. The yield stress of solutions was zero for almost all concentrations. The yield stress is defined as the stress that must be applied to a sample before it begins to deform or flow [60]. Below the yield stress, the sample will deform elastically; above the yield stress, the sample will flow like a liquid (plastic deformation). The zero value of the prepared solutions indicates that they flow under the slightest stress, which is a characteristic behavior of Newtonian fluids. These results imply that the studied solutions have Newtonian characteristics at low shear rates. The η_∞_ derived from the Sisko model was approximately zero at low concentration (1.25% *w*/*v*) and shifted to negative values with increasing the solution’s concentration (2.5% and 4% *w*/*v*). The obtained negative values have no physical meaning and could be attributed to model limitations and also to the shear-thinning characteristics of the prepared solutions, which result in zero viscosity at very high shear rates [61,62].

**Figure 4:**
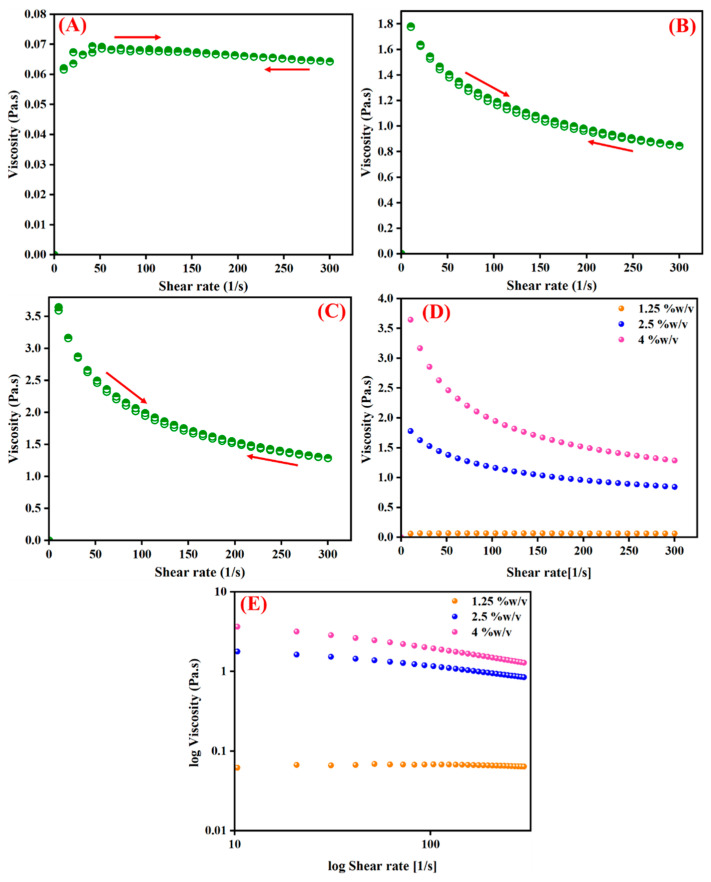
Viscosity against shear rate forward and backward rheograms of the chitosan solutions at (**A**) 1.25% *w*/*v*, (**B**) 2.5% *w*/*v*, and (**C**) 4% *w*/*v* concentrations. The (**D**) comparative and (**E**) double-logarithmic plots of forward viscosity-shear rate profiles of chitosan solutions at different concentrations.

The rheological characteristics of polymeric solutions could also be studied by establishing viscosity vs. shear rate rheograms. To this end, the respective viscosity-shear rate rheograms of chitosan solutions at varying concentrations were recorded, and the results are depicted in Figure 4A–C. From the results, the viscosity of the solutions significantly increases with an increase in the chitosan amount. Another important point is that all prepared solutions exhibited non-Newtonian shear-thinning properties and the pseudoplasticity characteristics of the solutions enhanced with increasing concentration and shear rate. The shear thinning behavior of polymeric solutions stems from enhanced molecular chain alignment caused by high shear stress or rate [63]. In other words, the macroscopic viscosity of the fluid system decreases due to the reduction in flow resistance between the oriented macromolecules. Moreover, as discussed in the previous sections, a narrow hysteresis loop starts to appear in the respective rheograms of all solutions, which is more pronounced at higher concentrations and shear rates.

The comparative and double logarithmic forward viscosity vs. shear rate plots of the solutions at different concentrations were extracted from their respective viscoelastic rheograms and depicted in Figure 4D,E. This figure demonstrates that the apparent viscosity of all solutions increases proportionally as the total concentration of chitosan is raised. However, as mentioned above, the viscosity of solutions showed shear-thinning characteristics, especially at higher concentrations, and decreased with increasing shear rate. The observed progressive decrease in the shear viscosity is associated with a degree of internal friction in the system, which increases with the shear rate caused by the effects of orientation or disentanglement of the chitosan macromolecules. No further change in the molecular shape or in the degree of entanglement is reflected in any change in viscosity.

**Figure 5:**
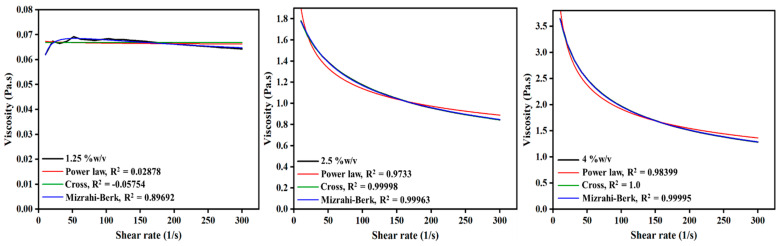
The curve fitting diagrams of chitosan solutions at different concentrations (1.25%, 2.5%, and 4% *w*/*v*) for the power law, Cross, and Mizrahi–Berk models.

In the typical viscosity-shear rate rheograms of polymeric solutions, two distinct regions with different rheological behaviors could be considered. (A) Zero-shear viscosity (η_0_) region at very low shear rates. This region provides information about the structure-function relation of biopolymeric systems. (B) Power-law region that is usually observed at higher shear rates and followed by a plateau region at very high shear rates (η_∞_). The power-law region gives information about the shear rate-dependent properties of formulations, which are suitable for process optimization and sensory characterization of polymeric solutions [64,65]. A linear relationship between viscosity and shear rate on a log-log plot is considered a good indicator for polymeric solutions that obey the power law models. Such plots that presented earlier in Figure 4E for the prepared chitosan solutions at different concentrations clearly indicate a linear relationship between viscosity and shear rate for the resultant solutions and that the prepared solutions obey the power-law model. In this sense, the viscosity profiles of different concentrations of chitosan solutions were further analyzed using the fitting process of their forward apparent viscosity profiles to power-law, cross, and Mizrahi–Berk models (Equations (4)–(6)). The results for each solution, along with the determination coefficient (R^2^) of the studied models. are presented in Figure 5. Table 2 provides a summary of the numerical values for the model’s parameters derived through the curve-fitting process using the double-logarithmic method.

From the R^2^ values in Figure 5, it is evident that the power law and cross models failed to fit the rheological data of the 1.25% *w*/*v* chitosan solution (resulting in very low and negative R^2^ values), while only a moderate correlation was obtained between the Mizrahi–Berk model and the experimental data. This could be attributed to the more Newtonian or semi-Newtonian behavior of chitosan solutions at lower concentrations. However, the R^2^ values of all studied models improved at higher concentrations of chitosan, indicating a good correlation between the three studied models and the experimental data. This implies a shear-thinning behavior for these solutions, which ideally obey the power-law model. Therefore, the power law, cross, and Mizrahi–Berk models are suitable models for estimating and determining the rheological behavior of chitosan solutions only at higher concentrations. From Table 2, the consistency index (K and K_M_) of the power law and Mizrahi–Berk models consistently increased with augmenting the solution’s concentration, while K_M_ produced negative values that render no physical meaning. The power law index of the prepared solutions steadily increased for the power law model. However, in the case of the cross model, this parameter initially increased with increasing concentration from 1.25% to 2.5% *w*/*v* and then decreased slightly for 4% *w*/*v* solutions. The power law index of the Mizrahi–Berk model exhibited an opposite trend to that of the power law model, steadily declining with increasing chitosan concentration. The *η*_∞_ values obtained from the cross and Mizrahi–Berk models also showed a similar trend with the power law index. However, the zero-shear viscosity of solutions in the cross model increased in a concentration-dependent manner. In conclusion, all prepared solutions exhibited elevated consistency at higher concentrations, with a pronounced shear-thinning behavior at higher shear rates and chitosan amounts in the solutions.

## 7. Dynamic Measurements of Viscoelastic Properties

**Figure 6:**
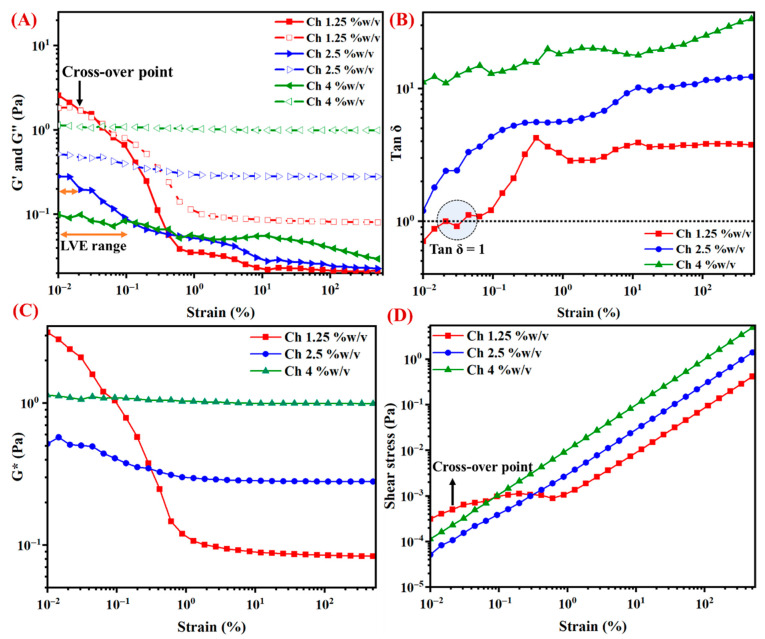
The variation in (**A**) storage and loss moduli, (**B**) damping factor, (**C**) complex modulus, and (**D**) shear stress profiles of chitosan solutions as a function of shear strain at different concentrations (1.25%, 2.5%, and 4% *w*/*v*). G′ (closed symbols) and G″ (open symbols).

To further investigate the rheological properties of chitosan solutions at different concentrations, the prepared solutions were subjected to dynamic and oscillatory rheological measurements to attain more information about the processing characteristics and structure–function properties of the resultant solutions. Figure 6A displays the variation in G′ and G″ of solutions in relation to shear strain at different chitosan concentrations. The strain sweep tests were conducted at strain amplitudes ranging from 0.01 to 500%, maintaining a constant frequency of 1 Hz and a temperature of 24 °C on the prepared chitosan solutions. This diagram could be used to determine the LVE region of polymeric solutions, where the storage modulus (G′) and loss modulus (G″) remain unaffected by the applied strain until a critical deformation point is reached [66,67]. In this region, rheological tests can be carried out without destroying the structure of the sample. Referring to Figure 6A, we were unable to observe an LVE range in the case of the 1.25% *w*/*v* chitosan solution and it may be located at very low shear strain values, which were not recorded in this study. However, for the 2.5% *w*/*v* solution, a narrow LVE range could be identified at lower shear strain values, which is more extended for the 4% *w*/*v* chitosan solution (indicated by a double arrow symbol). Therefore, we can conclude that increasing the solution concentration may result in a more extensive LVE range and shift the LVE region to higher shear strain values. This could be attributed to the higher interaction and hydrogen bonding of polymeric molecules at higher concentrations, which keeps the structure undisturbed for a more extended period. This phenomenon confirm the conclusion from the higher consistency index of chitosan solutions at higher concentrations obtained in previous sections. At the end of the LVE region, G′ starts to decline with increasing shear strain, indicating a significant distribution in the structure of polymeric components. The plateau value of G′ in the LVE region is a measure of the rigidity of the sample at rest, while the G″ value represents the viscosity of the unsheared sample. Besides the LVE region, the magnitude and ratio of dynamic moduli provide valuable information about the characteristics of the samples. In this respect, if G′ > G″, the sample behaves more like a viscoelastic solid, while for G″ > G′, it behaves as a viscoelastic fluid [68,69]. From Figure 6A, it is evident that the chitosan solutions at lower concentrations and shear strains behave like viscoelastic solids (elastic behavior). However, at higher concentrations and shear strains, they represent viscoelastic fluid characteristics (predominant gel structure). Moreover, the G″ profiles of all prepared solutions were almost constant in most regions of the diagram, especially at higher shear strains, while G′ profiles exhibited a great extent of fluctuations in all studied ranges of shear strains. It is evident that this fluctuation is higher for low concentrations of chitosan solutions, indicating their lower consistency and integrity. The crossover point of G′ and G″ implies that with increasing shear strain, the microstructure of the polymeric network expands and the gel state of the solutions converts to a semi-liquid state [70]. Among the prepared solutions, only the crossover point of the 1.25% *w*/*v* solution was observed within the studied shear strain range and it seems that the crossover points of higher concentration solutions of chitosan are located at shear strains lower than 0.01%. These observations could be explained by the high viscoelastic behavior of chitosan solutions, even at lower concentrations, which causes extensive swelling of the polymeric structure and chain entanglement in the random polymeric chains in the solutions [70,71,72]. Finally, it should be mentioned that the higher concentrations of chitosan solutions lead to higher differences between the G′ and G″ moduli, indicating their more pronounced and predominant viscous behavior (G″ > G′).

The non-linear viscoelastic properties of polymeric solutions can be revealed by assessing the damping or loss factor (Tan δ = G″/G′) shift in solutions over dynamic rheological tests. In general, the phase shift angle (δ) varies from 0° to 90° for the viscoelastic solutions and Tan δ varies from 0 to 1 dimensionless. For ideal elastic behavior, δ = 0°, i.e., Tan δ = 0, and for ideal viscous behavior δ = 90°, i.e., tan δ = ∞ [38]. The corresponding loss factor variation in the prepared solutions with amplitude strain is shown in Figure 6B. It can be seen that the damping factor of the solutions increases with an increase in shear strain and the solution’s concentration. However, this increase in the damping factor of formulations was more pronounced at lower shear strains, especially for low-concentration solutions (1.25% and 2.5% *w*/*v*), and became less affected at higher strains. Moreover, only in the case of 1.25% *w*/*v* chitosan solution, the Tan δ = 1 was observed at about 0.0211% amplitude strain (specified with a dashed circle), indicating more viscous behavior of the chitosan solution at higher concentrations and shear strains.

The complex modulus of solutions was calculated from their corresponding elastic and viscous modulus data sets, and the results are illustrated in Figure 6C. The complex modulus of polymeric solutions is defined as the ratio between the total stress and the deformation to which the material is subjected at a given frequency [73]. According to the results, the complex modulus of the prepared solutions declined with increasing shear strain and increased with higher chitosan concentrations. However, the complex modulus of the 2.5% and 4% *w*/*v* solutions showed little dependency on shear strain (slightly decreased with increasing shear strain) and almost remained constant at the studied amplitude strain range (especially for 4% *w*/*v* solution). This implies higher resistance to deformation of chitosan solutions at higher concentrations. In contrast, the complex modulus of 1.25% *w*/*v* chitosan abruptly decreased from higher values and reached a plateau state at higher shear strains.

The shear stress profiles of chitosan solutions at different concentrations were plotted as a function of shear strain and illustrated in Figure 6D. It can be observed that the shear stress of solutions elevated linearly with increasing shear strain. However, the lowest concentration solution of chitosan (1.25% *w*/*v*) exhibited a non-linear relationship at lower shear strains, which increased slightly in this region and then decreased to lower values, showing a linear dependence at higher shear strains. This may be attributed to the unstable elastic network of solutions with lower chitosan contents, resulting from insufficient molecular and secondary interactions between polymeric molecules, as observed in the storage, loss, and complex moduli profiles of the solutions in Figure 6A,C. A similar trend was also observed for low concentrations of other polymeric materials, such as PVA in Refs. [44,47,74,75]. Moreover, a concentration-dependent manner was observed for the prepared solutions at the studied concentrations, in which high-concentration solutions of chitosan produced higher shear stress profiles than those with lower concentrations. The flow-point stress of solutions could be determined through the crossover point of dynamic moduli, where G′ = G″. After this point, fluidization occurs and the solutions behave like a fluid [76]. By referring to Figure 6A, the crossover point only happened for 1.25% *w*/*v* solution of chitosan, and in the case of higher concentrations of chitosan solutions, no crossover point was detected. The respective strain amplitude and shear stress of this point for the 1.25% *w*/*v* solution of chitosan were 0.0211% and 5.06 × 10^−4^, respectively.

**Figure 7:**
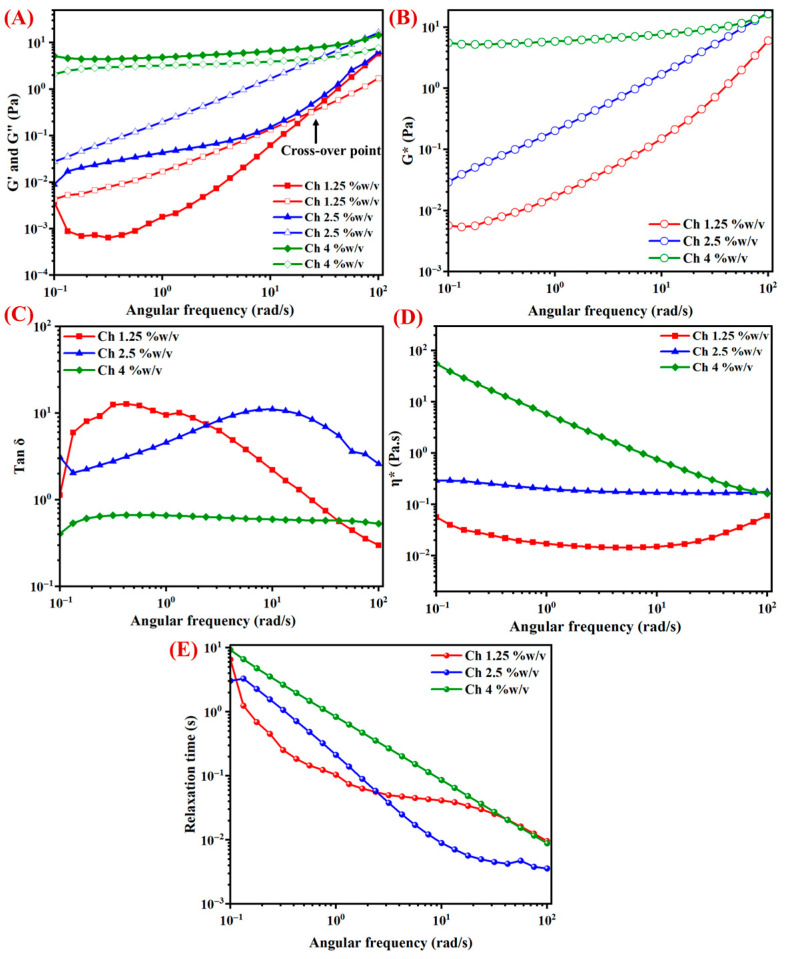
Dynamic rheological characteristics of chitosan solutions at different concentrations against angular frequency. (**A**) dynamic moduli, (**B**) complex modulus, (**C**) damping or loss factor, (**D**) complex viscosity, and (**E**) relaxation time. G′ (closed symbols) and G″ (open symbols).

The variation in dynamic moduli versus angular frequency of chitosan solutions at different concentrations is shown in Figure 7A. During the frequency sweep tests, the frequency was varied from 0.1 to 100 rad/s at a fixed 10% shear strain, where both dynamic moduli are relatively constant. It can be seen that at lower concentrations of chitosan solutions (1.25% and 2.5% *w*/*v*), both G′ and G″ increase strongly with increasing angular frequency (approximately 2–3 orders of magnitude). However, increasing the concentration to 4% *w*/*v* resulted in almost constant G′ and G″ profiles across the entire studied angular frequency range for the resultant solution. In addition, for the 1.25% *w*/*v* chitosan solution, G″ was higher than G′ at low frequencies (liquid-like behavior) and increased steadily until the crossover point at higher frequencies, where G′ exceeded G″ (gel-like behavior). By increasing the solution’s concentration to 2.5% *w*/*v*, a dominant viscous behavior was recorded throughout the entire frequency range. However, as shown in Figure 7A, the G′ approaches G″ at high frequencies for this solution, as for the 1.25% *w*/*v* solution. Further increasing the solution concentration to 4% *w*/*v* resulted in a pure gel-like behavior, and little difference was observed between the G′ and G″ profiles of the 4% *w*/*v* solution compared with those of lower-concentration solutions (1.25% and 2.5% *w*/*v*). Regarding these observations, the greater differences between G′ and G″ in 1.25% and 2.5% *w*/*v* solutions occurred at lower and intermediate frequencies. These results suggest that a critical concentration is required to establish a stable gel network from chitosan solutions, and that lower concentrations result in viscous behavior in the resultant solutions. Moreover, despite approaching the two dynamic moduli at higher frequencies and concentrations (2.5% and 4% *w*/*v*), no crossover point was observed for these solutions. Similar results were also reported in previous studies to those obtained in this study. For example, in the study conducted by Torres et al. using 2.5% and 3% *w*/*v* chitosan solution (85% DD) in 3% (*v*/*v*) of acetic acid, a viscous behavior (G″ > G′) was observed for the 2.5% *w*/*v* solution. In comparison, the 3.5% *w*/*v* solution exhibited almost a solid-like behavior over the studied frequency range (1–100 1/s) [77]. In another study by Rwei et al., the frequency sweep results of chitosan solutions (2 × 10^5^ and 88% DD) with 5, 8.5, 9, and 12% wt. concentrations exhibited a viscous-like behavior at lower concentrations (5% and 8.5%wt.), while a pure solid-like behavior was dominant at higher concentrations (9% and 12%wt.), a trend that was also observed in our study for medium molecular weight chitosan with 75–85% DD at 1.25–4% *w*/*v* concentrations [78]. The results of these studies are in good agreement with the findings of the present study.

Figure 7B displays the complex modulus of chitosan solutions at different concentrations against angular frequency. According to the results, the complex modulus of 1.25% and 2.5% *w*/*v* solutions steadily increased with increasing angular frequency. However, the 4% *w*/*v* solutions exhibited a relatively constant profile over the studied frequency range. In agreement with the results from the dynamic moduli of solutions in Figure 7A, the complex modulus of the chitosan solutions increased with concentration and higher concentrations exhibited higher complex moduli. According to these observations, the 4% *w*/*v* chitosan solution exhibited greater viscoelastic properties and resistance to deformation over the studied frequency range than solutions with lower concentrations.

The damping factor or loss factor of the prepared solutions in Figure 7C exhibited a drastic variation in response to angular frequency. From this figure, the low concentrations of chitosan solutions (1.25% and 2.5% *w*/*v*) displayed almost opposite trends throughout the studied angular frequency range. In this respect, the damping factor of the 1.25% *w*/*v* chitosan solution was higher at lower frequencies (Tan δ = 12.5, δ = 85.42°), which drastically decreased with increasing angular frequency (Tan δ = 0.299, δ = 16.64°). For 2.5% *w*/*v* chitosan solution, the damping factor was low at low frequencies (Tan δ = 0.133, δ = 7.58°) and increased to a peak value with increasing the angular frequency (Tan δ = 10.6, δ = 84.61°) and then decreased to lower values at very high frequencies (Tan δ = 2.6, δ = 68.96°). Consistent with its dynamic moduli, the damping factor of the 4% *w*/*v* solution was approximately constant over the studied angular frequency range (Tan δ = 3.16, δ = 72.44°). Accordingly, the 1.25% and 2.5% *w*/*v* chitosan solutions exhibited both elastic (Tan δ < 1) and viscous behavior (Tan δ > 1) based on angular frequency variation, while the 4% *w*/*v* solution only displayed elastic behavior. Therefore, the dynamic moduli and damping factor of chitosan solutions depend strongly on their final and practical concentration.

The frequency-dependent viscosity function, termed complex viscosity (η*), of solutions was determined during the forced harmonic oscillation of shear stress, with frequency ranges from 0.1 to 100 rad/s, and the results are presented in Figure 7D. The complex viscosity gives information about the viscous characteristics, stiffness elasticity, and shear-thinning behavior of the polymeric solutions. From Figure 7D, the 4% *w*/*v* chitosan solution exhibits high complex viscosity (55.6 Pa.s) at low frequencies, which decreases drastically to 0.164 Pa.s (the value of the 2.5% *w*/*v* solution ≈ 0.176 Pa.s) with the rise in angular frequency. The 2.5% *w*/*v* solution of chitosan showed only a slight decrease with increasing frequency and remained almost constant over the studied angular frequency range. In the case of the 1.25% *w*/*v* solution, the η* steadily decreased by increasing the angular frequency, followed by an increase at higher frequencies. The reduction in η* with increasing angular frequency, as observed for almost all solutions, indicates a non-Newtonian trend and is attributed to the shear-thinning effect of the formulations [79,80,81].

To further analyze the viscoelastic properties of chitosan solutions under dynamic shear, the relaxation time (λ) of the solutions was investigated. According to previous studies, the pseudostructures of polymeric molecules in the solution state by physical association or aggregation, significantly influence their relaxation behavior. In this sense, polymeric systems that exhibit a degree of molecular order or physical structure tend to display longer relaxation times. Consequently, the relaxation time of polymeric systems largely depends on their solution state, whether in a sol or a gel state. Figure 7E displays the relaxation time of chitosan solutions at varying concentrations, as calculated using Equation (8). The results indicate that the relaxation time of all solutions decreases with increasing frequency, a trend attributed to shear forces preventing polymeric chains from having sufficient time to rearrange at higher shear frequencies [45,47,48]. Moreover, the 4% *w*/*v* chitosan solution revealed the highest relaxation time than the other two solutions with lower concentrations (1.25% and 2.5% *w*/*v*). These results were expected, since the 4% *w*/*v* solution showed higher dynamic and complex moduli, and the lowest damping factor among the solutions in the Section 6. Accordingly, this solution possesses considerable molecular order or physical structure, referring to a gel-state structure. A similar trend was also observed for the 1.25% *w*/*v* chitosan solution at elevated frequencies, indicating its elastic behavior in this region. However, a viscous-like behavior was observed for this solution at lower frequencies. In the case of the 2.5% *w*/*v* solution, a pure viscous-like behavior was observed over all studied frequency ranges. Based on these observations, we can conclude that the viscoelastic behavior of chitosan solutions is strongly affected by their total concentration. In this context, the lowest concentration of chitosan (1.25% *w*/*v*) exhibited both elastic and viscous behavior depending on the frequency. On the other hand, the 2.5% *w*/*v* solution displayed pure viscous behavior over the entire studied frequency range. By increasing the concentration to a critical point (4% *w*/*v*), pure elastic behavior is observed. Similar results have also been reported in previous studies for other polymeric solutions, such as polyvinyl alcohol (PVA) solutions [44,47,74,75]. The relaxation time of polymeric solutions, as generally understood, is influenced by the molecular weight of the polymer and the concentration of the solution. Several studies have reported that increases in both molecular weight and concentration lead to stronger intermolecular interactions and greater molecular aggregation, which subsequently limit the motion of molecular chains, thereby increasing the relaxation time. Moreover, Junfang Li et al. analyzed the relaxation behavior of polymeric solutions using dynamic laser light scattering (LLS). They emphasized that at higher polymer concentrations, a slow relaxation mode becomes prominent in concentrated regions such as melts and gels, where polymer chains are crosslinked rather than simply entangled. This slow relaxation mode has similarly been observed in solutions containing charged macromolecules, both aqueous and nonaqueous. Ultimately, the authors concluded that this slow relaxation mode can be qualitatively attributed to the restricted movement of interacting chains. This remains true despite variations in the nature of polymeric interactions, ranging from weak segment-segment interactions in poor solvents to strong electrostatic interactions among polyelectrolyte chains, and even to chemical crosslinking within gel networks [82]. Therefore, these differences in various polymeric solutions could stem from their different structural characteristics (molecular weight, hydrogen bonding, hydrophobic interactions, etc.) and solution properties (sol and gel). Accordingly, polymers with higher molecular weights and extensive primary and secondary interactions (hydrogen bonding, hydrophobic interactions, etc.), especially in the gel state, could lead to slower polymer chain motions and, thereby, lower relaxation times.

**Figure 8:**
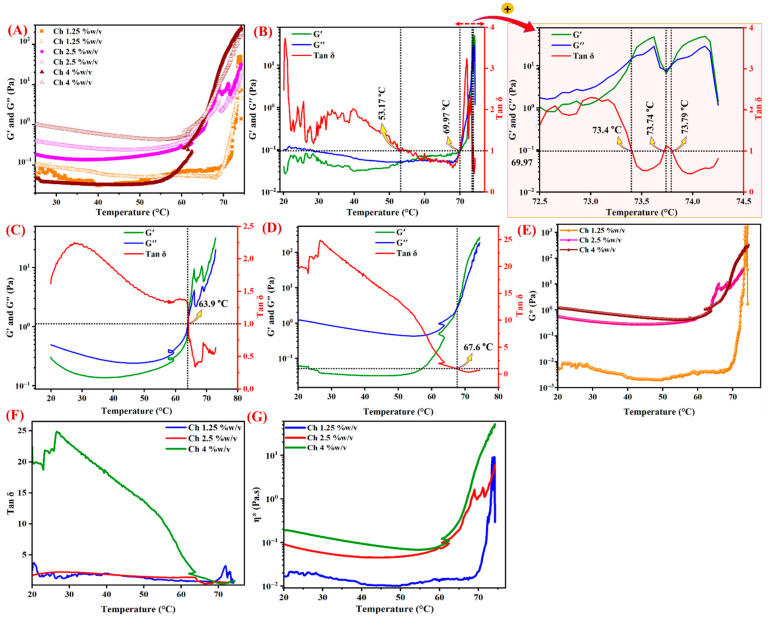
Evolution of dynamic rheological characteristics of chitosan solutions at different concentrations with temperature at a heating rate of 5 °C/min and frequency of 1 Hz. (**A**–**D**) dynamic moduli, (**E**) complex modulus, (**F**) damping factor, and (**G**) complex viscosity. G′ (closed symbols) and G″ (open symbols).

The temperature-dependent dynamic rheological characteristics of polymeric solutions are important for their processing and the development of various end products with desired mechanical and thermogelling properties. Given its high importance, dynamic rheological experiments on chitosan solutions at different concentrations are carried out as a function of temperature. Figure 8A–G display the various rheological properties of the prepared chitosan solutions in terms of dynamic moduli, complex modulus, damping factor, and complex viscosity obtained at a constant amplitude strain and frequency of 10% and 1 rad/s, respectively. The temperature was increased continuously from 20 to 75 °C at a rate of 5 °C/min. The variation in G′ and G″ profiles of chitosan solutions in Figure 8A reflects a strong dependence of the prepared solution on variable temperature, especially at higher temperatures. The storage modulus is related to the solid-like element of the rheological behavior; and therefore, it is low at the solution stage but increases significantly at the gelation temperature. Gelation temperature is a crossover point in the rheological diagrams of polymeric solutions in which a sol/gel transition occurs and G′ becomes equal with G″ (Tan δ = 1). The gelation temperature provides a valuable standpoint for evaluating the thermosensitive properties of (hydro)gel-forming polymeric solutions. From Figure 8A, the G′ and G″ profiles of solutions were almost constant (slightly decreased at low temperatures) and abruptly increased at higher temperatures. Accordingly, the obtained diagrams could be divided into two regions. In the first region, G′ was lower than G″ for all formulations, indicating their highly viscous and liquid-like behavior at lower temperatures. Moreover, it is evident that increasing the solution’s concentration increases the storage and loss moduli of the solutions and also results in higher differences between the two moduli. With further increasing the temperature, both moduli began to increase abruptly, and the gelation temperature was reached. This sharp increase in G′ could be attributed to the partial formation of chitosan clusters through hydrophobic interactions. However, at the adjacent of the gelation temperature, a few chitosan chains form polymeric clusters [83,84]. But these clusters are too minuscule to create a completely mature gel network. After the gelation temperature, G′ becomes greater than G″ with increasing temperature and forms an elastic gel network. Regarding the abrupt increase in the dynamic moduli at higher temperatures, similar results were also reported by Parparita et al. on PVA/Ch composite films using dynamic mechanical analysis (DMA) and by Ripoll et al. for xanthan gum-based coating solutions enriched with phenolic mango peel extracts. The results of these studies indicated a marked increase in dynamic moduli of polysaccharide systems at approximately 45–70 °C, which aligns with the rise in moduli observed in our study at higher temperatures [85,86].

**Scheme 1:**
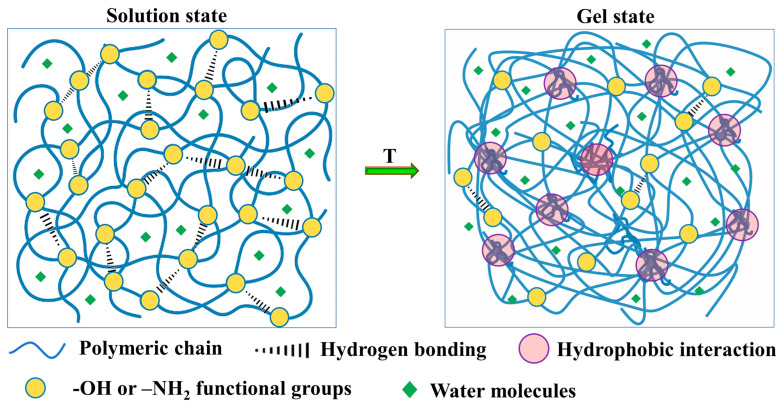
Gel formation mechanism of chitosan solutions at high temperature.

For a better investigation of the gelation behavior of solutions, the diagrams of dynamic moduli and damping factor of solutions as a function of temperature were plotted separately for each solution, and the results are presented in Figure 8B–D. In these rheograms, the crossover point of G′ and G″ moduli represents the gelation temperature of solutions (specified by the crossover vertical and horizontal dotted lines) at which Tan δ becomes unity. The 2.5% and 4% *w*/*v* chitosan solutions exhibited a single gelation temperature in their respective rheograms at 63.9 °C and 67.6 °C, respectively (Figure 8C,D). However, the 1.25% *w*/*v* chitosan solution showed several gelation temperatures (Figure 6B) at 53.17, 69.97, 73.4, 73.74, and 73.79 °C, respectively. In these temperatures, the G′ and G″ moduli profiles of solutions frequently change over the studied temperature range, especially at higher temperatures. These observations could be related to the formation of a loose gel structure in the 1.25% *w*/*v* solution at higher temperatures, due to the inadequate number of chitosan molecules in this solution required for the gel formation process. By considering the main gelation temperature of 1.25% *w*/*v* solution at 53.17 °C, the gelation temperature of solutions increased by increasing the concentrations of solutions (to 63.9 and 67.6 °C for 2.5% and 4% *w*/*v* solutions, respectively). These results are in good agreement with the generally accepted gelation mechanism. In this sense, the higher content of chitosan can produce more hydrogen bonding and other secondary interactions, which need a higher gelation temperature to reduce the hydrogen bonds and promote the gel formation through hydrophobic interactions (Scheme 1) [87,88].

The complex modulus of the chitosan solutions as a function of temperature was determined from their corresponding storage and loss moduli, and the results are presented in Figure 8E. It can be seen that the complex modulus of the solutions is less affected at low temperatures and slightly decreases with increasing temperature. However, increasing the temperature to above 60 °C resulted in a significant increase in the complex modulus of solutions. It is worth noting that the complex modulus of solutions is more affected by their viscous modulus (showing a similar trend with G″ profiles in Figure 8A), especially at higher concentrations and lower temperatures, indicating their dominant liquid-like behavior at these concentrations. However, at low concentrations and elevated temperatures, a more solid-like behavior (higher G′ values) was observed for all solutions, implying the formation of a polymeric gel network. Moreover, the complex modulus of chitosan solutions exhibited a considerable dependency on the concentration of the solutions and significantly increased with increasing concentration.

The comparative damping factors of the studied solutions in relation to temperature are represented in Figure 8F. According to this diagram and in agreement with the results obtained above for dynamic moduli, the damping factors of chitosan solutions augmented by increasing solution’s concentration and declined by increasing temperature. These results imply more viscous characteristics and dissipative capacity of chitosan solutions at high concentrations and lower temperatures. Moreover, the damping factor of 4% *w*/*v* of chitosan exhibited a considerable dependency on temperature than other solutions, especially at lower temperatures, which may be related to its more viscous behavior at these temperatures.

The complex viscosity profiles of chitosan solutions against temperature are displayed in Figure 8G. It can be seen that the complex viscosity of solutions reveals similar trends with their dynamic moduli at both lower and higher temperatures, reflecting the concentration- and temperature-dependent variation in this parameter for the resultant solutions. In this regard, the complex viscosity of the solution decreased with increasing temperature until a critical value and then increased suddenly at higher temperatures. The latter could be attributed to their gel-forming characteristics or to increase in solution concentrations resulting from solvent (water) loss during experiments at elevated temperatures. Agreeing with the first conjecture, the breaking of inter- and intra-molecular hydrogen bonds in polymeric molecules start at higher temperatures and encourages the gel formation process via the establishment of more hydrophobic interactions (Scheme 1) [87,88].

**Figure 9:**
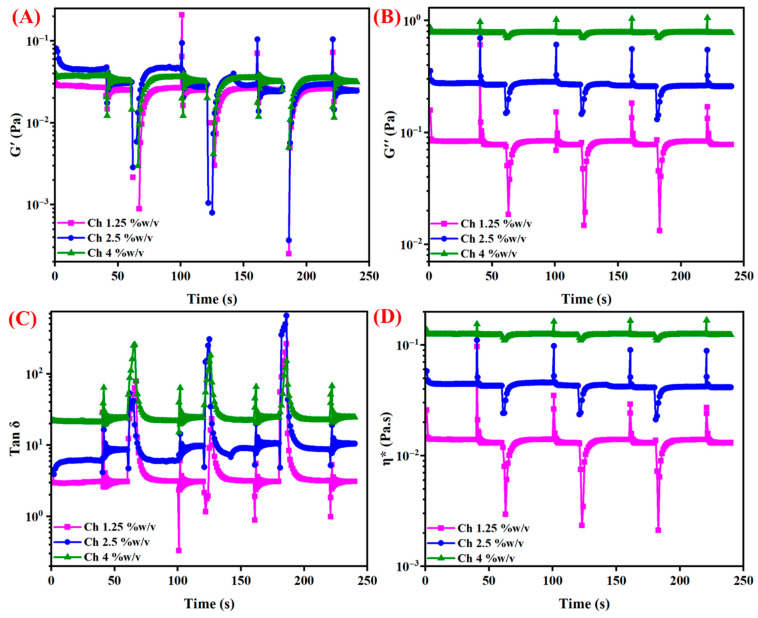
The recoverability of chitosan solutions at different concentrations (1.25%, 2.5%, and 4% *w*/*v*) by cyclic strain measurements at high (150%) and low (10%) strains, showing storage modulus (**A**), loss modulus (**B**), damping factor (**C**), and complex viscosity (**D**) in four cycles. The measurements were performed at 24 °C.

In the next step, cyclic strain measurements (each cycle was 10 s) were conducted to investigate the recoverability of chitosan solutions at different concentrations in terms of storage and loss moduli, damping factor, and complex viscosity. The corresponding rheograms of each solution are presented in Figure 9A–D. From the results, solutions containing higher amounts of chitosan resulted in much higher G′, G″, Tan δ, and η*. The larger difference among the various concentrations of chitosan solutions for the studied rheological characteristics was observed in G″ and η*, while the G′ and Tan δ profiles of the solutions were pretty close to each other. Moreover, it can be seen that the chitosan solutions with higher concentrations exhibited highly stable profiles, with almost constant values recorded for G′, G″, and η* in each cycle, indicating their desired capability to recover their structure rapidly. However, the low-concentration solution of chitosan (1.25% *w*/*v*) showed a large variation after each cycle, and its corresponding storage and loss moduli, as well as complex viscosity profiles, decreased significantly after the first cycle. However, in the case of the damping factor, the highest and lowest variations were observed for 2.5% and 4% *w*/*v* chitosan solutions, respectively, and their values increased significantly over time after the first cycle. From the obtained results, it is reasonable to conclude that the higher concentration of chitosan solutions displays better recoverability than their lower concentration solutions. This could be related to the larger number of chitosan molecules, and thereby, the vast number of hydroxyl and amine functional groups in these solutions, and also lower protonation of amine groups at the same concentration of acetic acids, which facilitates hydrogen bonding and other intramolecular interactions between polymeric molecules. In other words, in lower concentrations of chitosan solutions, the electrostatic repulsion forces between protonated amines of chitosan molecules (higher than concentrated solutions) hinder them from effectively approaching together and recover their original structure. Moreover, the smaller number of polymeric molecules in these formulations, hinders them from finding and reaching together in order to establish effective hydrogen bonds and other intramolecular interactions. These findings also confirm the liquid-like viscous properties of chitosan solutions at elevated concentrations.

## 8. Conclusions

In the present study, the rheological characteristics of chitosan solutions at different concentrations were investigated using various rheological measurements and correlated with different mathematical models. Based on the results, the shear stress and shear viscosity of chitosan solutions were increased in a concentration-dependent manner. Moreover, the higher concentrations of chitosan exhibited higher consistency and shear-thinning properties with minor thixotropic behavior, especially at higher shear rates. In shear stress–shear rate measurements, all studied models successfully correlated with the obtained data for all studied solutions. However, in viscosity–shear rate measurements, the studied Power law, Cross, and Mizrahi–Berk models showed a desired correlation only at medium and high concentrations of chitosan (2.5% and 4% *w*/*v*). Evaluating the dynamic viscoelastic properties of solutions through amplitude strain sweep tests revealed that the lower concentrations of chitosan exhibit elastic behavior at lower shear strains, while the higher concentrations showed predominantly viscous behavior over the entire shear strain range. During the frequency sweep measurements, the 1.25% and 2.5% *w*/*v* of chitosan solutions exhibited viscous-like behavior, while the 4% *w*/*v* solution showed solid-like behavior with nearly constant storage and loss modulus profiles over the studied frequency range. In the temperature sweep measurements, viscous-like behavior was dominant at lower temperatures, which resulted in elastic gel structures at higher temperatures. Assessing the recoverability of chitosan solutions through cyclic strain measurements revealed that the high-concentration solutions possess the desired capability to recover their structure. In summary, the results of this study revealed that the variation in chitosan solution concentrations strongly affects their rheological properties in terms of various steady and dynamic rheological characteristics. However, additional studies are required to fully elucidate the chitosan rheological properties, such as specifying the exact regimen of chitosan solutions at higher concentrations with different molecular weights, concentrations, acid types and amounts, DDs, etc., unraveling the reason for the irregularities observed in the temperature sweep tests, as well as the impact of other synthetic and natural polymers on the rheological properties of chitosan solutions.

## Figures and Tables

**Table 1:** The numerical results of the model fitting procedure using rheological models obtained in relation with forward rheograms of chitosan solutions at different concentrations tested at 25 °C.

Concentration (% *w*/*v*)	Ostwald–De Waele		Herschel–Bulkley			Sisko		
	K (Pa.s^n^)	n	*τ*_0_ (Pa)	K (Pa.s^n^)	n	K (Pa.s^n^)	*η*_∞_ (Pa.s)	n
1.25	0.084	0.95	0	0.084	0.95	0.084	0	0.95
2.5	4.29	0.72	0	4.33	0.72	6.99	−4.21	0.94
8	10.72	0.63	0	10.82	0.63	8.68	−1.68	0.73

**Table 2:** Numerical results of the power law, Cross, and Mizrahi–Berk model’s parameters obtained in relation with forward rheograms of chitosan solutions at different concentrations.

Concentration (% *w*/*v*)	Power Law		Cross				Mizrahi–Berk		
	K (Pa.s^n^)	n	*η*_∞_ (Pa.s)	η_0_ (Pa.s)	t (s^n^)	n	K_OM_ (Pa.s^n^)	K_M_ (Pa.s^n^)	n
1.25	0.068	0.005	0.071	0.063	0.027	0.0	−0.221	0.358	0.45
2.5	3.23	0.23	0.30	2.09	0.011	0.71	−2.51	3.40	0.30
4	7.98	0.31	0.26	5.04	0.025	0.65	−4.92	6.35	0.24

## Data Availability

The raw data supporting the conclusions of this article will be made available by the authors upon request.
